# Biofortification of Vegetables with Iodine and Molybdenum for Healthy Nutrition: A Controlled Trial

**DOI:** 10.3390/nu18010002

**Published:** 2025-12-19

**Authors:** Sara Baldassano, Luigi Di Rosa, Cristina Cortis, Alessia Cannizzaro, Antonino Salvatore Fiore, Leo Sabatino, Sonya Vasto, Patrizia Proia

**Affiliations:** 1 Department of Biological, Chemical and Pharmaceutical Sciences and Technologies, University of Palermo, 90139 Palermo, Italy; sara.baldassano@unipa.it (S.B.); luigi.dirosa@unipa.it (L.D.R.); alessia.cannizzaro03@community.unipa.it (A.C.); antoninosalvatore.fiore@community.unipa.it (A.S.F.); 2 Department of Human Sciences, Society and Heath, University of Cassino and Lazio Meridionale, 03043 Cassino, Italy; c.cortis@unicas.it; 3 Department of Agricultural, Food and Forest Sciences (SAAF), University of Palermo, 90128 Palermo, Italy; leo.sabatino@unipa.it; 4 Sport and Exercise Sciences Research Unit, Department of Psychological, Pedagogical and Educational Sciences, University of Palermo, 90128 Palermo, Italy; patrizia.proia@unipa.it

**Keywords:** micronutrients, metabolic, non-communicable disease, inflammation, sustainable food

## Abstract

**Background/Objectives**: Excessive sugar, fat, and salt intake heighten susceptibility to metabolic syndrome and other chronic metabolic conditions. Biofortification (i.e., enhancing the nutritional content of crops) emerges as a sustainable new approach to address dietary deficiencies. **Methods**: This study evaluated the impact of an acute nutritional intervention in a controlled, randomized, single-blind trial involving healthy adults aged 50–79 years, in late middle age and early older adulthood utilizing biofortified vegetables enriched with iodine and molybdenum, aimed to explore short-term biochemical responses to the consumption of iodine- and molybdenum-biofortified lettuce. The study was designed as a controlled dietary intervention including both a biofortified and a non-biofortified lettuce group, matched for handling and composition. It was powered to detect short-term biochemical responses, providing initial insights into the physiological impact of micronutrient biofortification. Dietary intake was carefully monitored throughout the 12-day period to control for confounding dietary effects. **Results**: The intervention was associated with decreased plasma levels of triglycerides, AST, and ALT, and increased plasma levels of HDL-cholesterol and the satiety hormone PYY, suggesting enhanced metabolic regulation. **Conclusions**: These biochemical markers reflect early metabolic adaptations that may inform future research on the metabolic impact of micronutrient biofortification. This study also highlights the potential of crop biofortification as a sustainable, strategy to enhance the nutrient density of vegetables within controlled dietary patterns.

## 1. Introduction

Diet plays a pivotal role in maintaining human health by supporting physiological functions and providing essential nutrients across the lifespan [1,2,3,4,5]. Modifying dietary habits, such as increasing the consumption of nutrient-rich foods and reducing the intake of unhealthy components, can significantly improve well-being across different population groups [6,7,8,9]. Conversely, excessive intake of sugar, saturated fat, and salt have been associated with increased risk of several chronic diseases, such as cardiovascular conditions, metabolic disorders, neurodegenerative diseases, and cancer [10,11,12,13,14,15]. In this context, agricultural biofortification has emerged as a promising strategy to enhance the nutritional quality of foods by increasing their content of essential vitamins and minerals. This approach is particularly relevant for addressing micronutrient deficiencies that may contribute to impaired metabolic regulation. In addition, biofortification connects agricultural innovation with human health outcomes, bridging the “farm-to-fork” continuum.

Emerging evidence suggests that trace elements such as iodine and molybdenum are involved in modulating pathways associated with metabolic and neurological processes [15,16,17]. Moreover, iodine and molybdenum intake has been linked to key metabolic processes, including bone homeostasis and iron metabolism; no established role in glycemic regulation has been demonstrated in humans [18,19,20].

The human body contains approximately 15–20 mg of iodine, primarily bound in thyroid hormones, with smaller amounts in tissues such as the mammary glands, gastric mucosa, and salivary glands [21]. The recommended dietary allowance (RDA) for iodine is 150 µg/day [21]. Iodine, a key component of thyroid hormones triiodothyronine (T3) and thyroxine (T4), is crucial for regulating metabolism [22]. Iodine, in the form of iodide, may possess antioxidant-like properties, partly due to its ability to act as a reducing agent [23]. Therefore, iodine has been proposed to exert antioxidant-like effects primarily in in vitro systems, because some in vitro and animal studies have suggested possible antioxidant-like effects of iodide [24,25]. However, evidence in humans remains limited [26,27] and inconclusive.

It is well known that iodine deficiency can lead to hypothyroidism, characterized by a reduced Basal Metabolic Rate (BMR) and impaired lipid metabolism [20]. This condition often results in weight gain and increased cholesterol levels due to decreased lipid breakdown. Conversely, excessive iodine intake can cause hyperthyroidism, leading to an increased BMR and excessive lipid catabolism, which may result in weight loss and muscle wasting [28]. Similarly, molybdenum acts as an essential cofactor for enzymes involved in detoxification, purine metabolism, and antioxidant defense (e.g., sulfite oxidase, xanthine oxidase) [29]. Molybdenum is found in legumes, grains, and animal organs because of its presence in soil and incorporation into the food chain [30]. It is rapidly excreted in urine [31], allowing for easy monitoring of intake. The RDA for molybdenum is 45 µg/day, with established biomonitoring equivalents (BEs) indicating toxicity at serum and urine levels of 31 µg/L and 7500 µg/L, respectively [32]. Aside from its role in detoxification and uric acid synthesis, molybdenum has been associated with a reduced risk of esophageal cancer [17] and may also influence oxidative and neurological processes [33]. Botchway and colleagues also discussed the antioxidant properties of molybdenum, highlighting its potential role in modulating redox balance and cellular protection mechanisms [33]. Molybdenum plays a role in maintaining lipid homeostasis, particularly through its involvement in the oxidative breakdown of fatty acids in the context of metabolic disorders; however, while some animal studies suggest effects on preventing lipid accumulation, this has not been demonstrated in humans. Furthermore, elevated xanthine oxidase activity is associated with liver pathologies such as non-alcoholic fatty liver disease (NAFLD) and hepatocellular carcinoma (HCC) [34]. In vitro, molybdenum particles showed mitochondrial protection, modulation of anti-inflammatory cytokines (TGF-β) and suppressing pro-inflammatory ones (TNF-α, interleukin (IL)-6, and IL-1β) [32,34].

Despite their established roles, the potential of targeted, food-based supplementation with iodine and molybdenum to modulate specific metabolic biomarkers in different populations remains underexplored. Most research focuses on deficiency states rather than optimal supplementation for health promotion. A significant research gap exists in understanding the acute, direct metabolic effects of consuming biofortified foods enriched with these micronutrients. Therefore, the objective of this study was to conduct an exploratory evaluation of short-term biochemical responses following consumption of iodine- and molybdenum-biofortified lettuce. The study did not aim to assess clinical outcomes or long-term disease prevention. More specifically, our study evaluated the impact of daily intake of iodine- and molybdenum-biofortified vegetables on biomarkers relevant to metabolic regulation, including lipid profiles, oxidative stress, and pro-inflammatory cytokines in comparison to the control diet for 12 days. Through this intervention, we will further explain the potential for targeted micronutrient supplementation (via biofortified foods) to influence endpoints relevant to metabolic health and micronutrient physiology [35,36].

## 2. Materials and Methods

### 2.1. Study Design

This study is part of the larger Nutri-Mo-Food project, registered at ClinicalTrials.gov under the approval number NCT04984746. This study adhered to the Declaration of Helsinki, was conducted in accordance with ethical guidelines and received approval from the Ethics Committee of the Palermo University Hospital (approval number 2/2020 AIFA, date of approval 19 February 2020).

The trials took place in the Nutrition, Age, and Bone (NABbio) laboratory of the Department of Biological, Chemical and Pharmaceutical Sciences and Technologies (STEBICEF) at the University of Palermo, under controlled conditions. During the first visit, participants signed an informed consent and underwent anthropometric, lifestyle and dietary intake assessments [37,38]. The study is a 12-day, controlled, single-blind, randomized nutritional intervention. Participants were masked and they did not know if they were receiving the biofortified or control lettuce. However, the personnel who grew, prepared, packaged the 200 g portions of lettuce were aware of which was which only the people measuring outcomes (e.g., taking blood, analyzing samples) did not know which group the samples came from. Investigators and medical staff were masked during data collection and analysis.

During the screening interview, participants were required to complete a health questionnaire, maintain a daily food diary, and provide lifestyle information to determine eligibility for inclusion (see Supplementary Materials). Individuals with a known history of blood-related dysfunctions, cardiac, gastrointestinal, or metabolic disorders, or recent viral infections were excluded from participation. Additionally, those using supplements (e.g., molybdenum and/or iodine), medications (including exogenous hormones for females), or topical products (e.g., CI 45430 molecule, *Fucus vesiculosus* and *Laminaria digitata* extract) for more than 6 weeks before the study onset were also excluded. Participants with known adverse or allergic reactions to lettuce consumption were also excluded. A summary of the eligibility criteria is provided in Table 1.

Eligible participants who provided informed consent underwent baseline hematological testing and were subsequently randomly allocated through a single-blind procedure to receive vegetables. More specifically, participants in the intervention group were provided with a total of 3 kg of either biofortified lettuce (Biofortified group) or control lettuce (Control group). This quantity was determined to ensure participants could reliably meet the daily consumption target of 200 g over the 12-day period (2.4 kg consumed), accounting for natural biological variation, necessary discarding of outer leaves, and cleaning-related preparation losses inherent when working with fresh vegetables.

### 2.2. Participants

**Figure 1:**
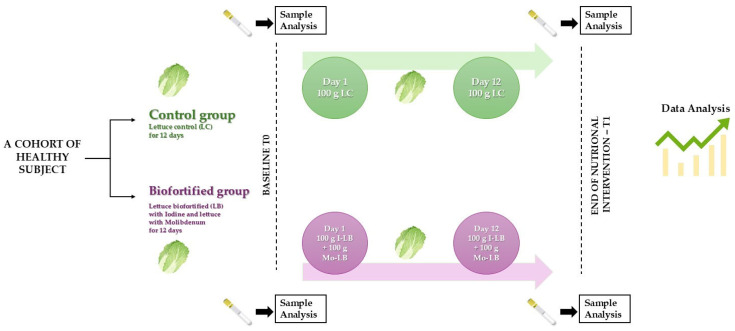
Overview of the experimental design. LC = lettuce control, LB = lettuce biofortified (100 g I-LB + 100 g Mo-LB).

**Figure 2:**
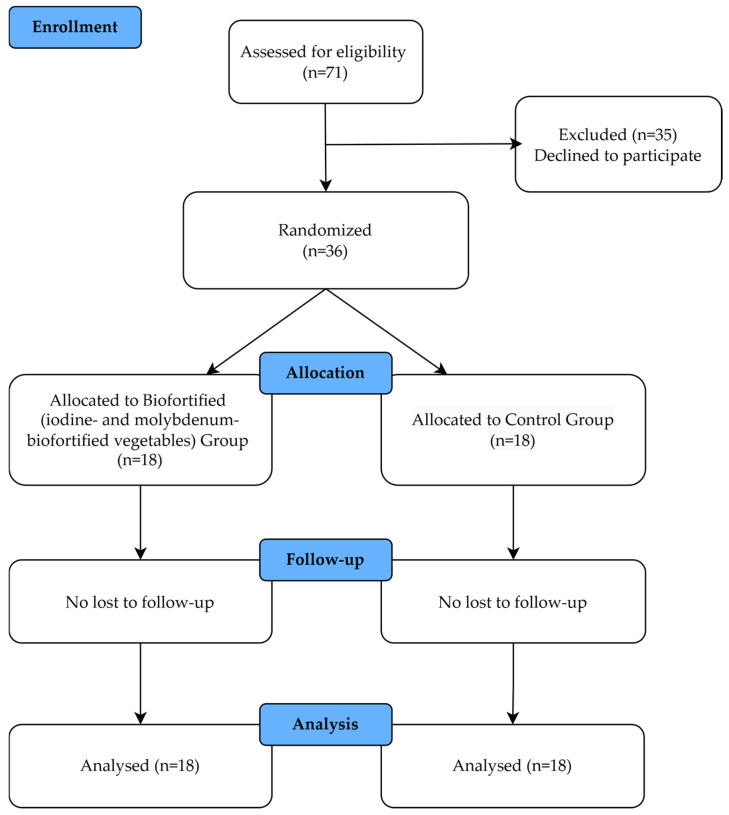
Overview of the experimental design by CONSORT diagram.

An a priori power analysis was performed based on previous studies involving hematological parameters [37,38]. Assuming a significance level (α) of 0.05 and a statistical power (1 − β) of 0.80, the minimum estimated sample size required to detect meaningful differences was eight participants per group (Biofortified and Control). To further reduce the risk of type II error and strengthen the robustness of secondary analyses, the final sample size was increased to eighteen participants per group. This ensured that the study was adequately powered to detect relevant short-term physiological effects. In addition, this sample size is consistent with previous [37,38] controlled-feeding trials evaluating acute metabolic responses to micronutrient-enriched foods, and the present study was specifically designed to investigate short-term biochemical adaptations rather than long-term clinical outcomes. The initial screening process evaluated 71 potential participants (age range 50–79 years, mean age 57.8 years) for eligibility based on the established inclusion and exclusion criteria. Following this screening, 35 withdrew and 36 eligible individuals (19 females and 17 males) were enrolled and subsequently randomized into two groups (18 participants per group): the Biofortified (iodine- and molybdenum-biofortified vegetables) group and the Control group that concluded the study (Figure 1 and Figure 2).

All participants were instructed by qualified nutritionists and physicians to maintain consistent dietary habits and lifestyle behaviors, including physical activity levels, throughout the study period. Participants received detailed guidance and examples on how to properly complete their food diaries. As outlined in the literature [20], food diaries were completed for 8 days prior to the intervention and continued throughout the 12-day study period. The records were used to monitor adherence, compliance, and any changes in food habits and lifestyle during the intervention. To minimize potential dietary confounding, participants’ total dietary intake was systematically monitored throughout the study. Daily food records were complemented by dietary recalls conducted by trained staff to verify accuracy and completeness. All dietary data were analyzed to confirm that no significant differences existed between groups in energy or micronutrient intake apart from the components provided through the experimental intervention. This procedure ensured that any observed outcomes could be attributed to the biofortified lettuce rather than differences in background diet. Physical activity (PA) during the 12-day intervention was monitored using the validated Single-Item Measure (SIM), a self-report instrument chosen to assess adherence to standard public health PA guidelines while minimizing participant burden. The SIM question administered was: “In the past week, on how many days have you done a total of 30 min or more of physical activity, which was enough to raise your breathing rate? (This may include sport, exercise, and brisk walking or cycling for recreation or to get to and from places but should not include housework or physical activity that may be part of your job)”. Participants provided a numerical response from 0 to 7 days. To capture the activity trajectory across the 12-day intervention with statistical rigor and avoid the temporal overlap inherent in administering the “past week” question daily, the SIM was administered at three key time points: Baseline (Pre-Intervention) on Day 1, immediately before the intervention began; Mid-Intervention on Day 7; and Post-Intervention on Day 13 (final data collection).

### 2.3. Procedures

The study was conducted as a single-blind randomized controlled trial, with participants, clinical staff, and outcome assessors blinded to group allocation throughout the study. Only horticultural personnel involved in the cultivation phase were aware of the fortification assignments; they had no contact with participants and no role in data collection or analysis. Sample assessment and data analysis were also performed under blinded conditions. Participants received approximately 3 kg of lettuce to ensure a daily intake of 200 g/day for 12 days. The extra quantity accounted for natural variation in leaf size, trimming of outer leaves, and minor preparation losses. Adherence was monitored through daily distribution logs and participant confirmation. Although weighed measurements of leftovers were not collected, participants were instructed to consume 200 g/day for the entire intervention period. The Control group received 200 g/day of lettuce grown under identical agronomic, handling, and post-harvest conditions as the experimental product, but without biofortification. The control lettuce contained only naturally occurring background levels of molybdenum and iodine (0.21 mg sodium molybdate and 0.27 mg potassium iodate per 100 g). The Biofortified group received 100 g/day of molybdenum-fortified lettuce and 100 g/day of iodine-fortified lettuce, for a total daily intake of 200 g over 12 days. Iodine enrichment was achieved by supplying potassium iodate (KIO_3_; CAS No. 7758-05), following the method described by Baldassano et al. [37]. Molybdenum enrichment was implemented through foliar spraying of sodium molybdate (Na_2_MoO_4_·2H_2_O; CAS No. 10102-40-6), according to the protocol described by Sabatino et al. [39]. Both compounds were purchased from Sigma-Aldrich/Merck (Steinheim, Germany). Foliar spraying was selected as the fortification method because it delivers the micronutrient directly onto the plant surface, whereas soil fortification is associated with highly variable absorption and limited control over the bioavailable fraction [40]. The biofortification procedure was the only difference between treatments, allowing observed effects to be specifically attributed to the consumption of biofortified lettuce. For consistency of reporting, elemental molybdenum and iodine concentrations were expressed as their corresponding salt equivalents (sodium molybdate and potassium iodate). The reported values of Na_2_MoO_4_ (8 mg/100 g) and KIO_3_ (1.21 mg/100 g) refer to the fortification solutions applied during cultivation and not to the concentrations present in the edible leaves, as incomplete foliar uptake and post-harvest losses occur during growth and washing.

Before (T0) and after (T1) the intervention, body composition (percentage of fat mass, muscle mass, visceral fat) and basal metabolic rate (kcal) were assessed using bioelectrical impedance analysis (InBody 320 Body Composition Analyzer, InBody Co., Amsterdam-Zuidoost, The Netherlands) [38]. Standing height (barefoot) and body weight were measured using a wall-mounted stadiometer (ADE MZ10042, ADE, Hamburg, Germany) and an electronic scale (Gima 27335, Zhongshan Transtek Electronics Co., Zhongshan, China) [38]. Body mass index (BMI) was calculated as weight (kg) divided by height squared (m^2^). Chest, waist, abdominal, and hip circumferences (cm) were measured using a flexible measuring tape (seca 201, Intermed SRL, Milan, Italy).

Venous blood samples were collected at T0 and T1 in the morning after an overnight fast to minimize circadian variation (Figure 1). Blood was collected into EDTA tubes for plasma analysis and into VACUETTE^®^ serum tubes for hematochemical analyses. Plasma samples were obtained by centrifugation at 1509× *g* for 10 min at 4 °C. Serum samples were allowed to clot for 30 min at room temperature before centrifugation. All analyses were performed using FDA-cleared and CE-marked methods at the Central Laboratory of the University Hospital of Palermo. Lipid profile parameters, including total cholesterol, triglycerides, low-density lipoprotein (LDL), and high-density lipoprotein (HDL), were measured using standard enzymatic methods. Liver enzymes alanine aminotransferase (ALT) and aspartate aminotransferase (AST) were also assessed. Thyroid function was evaluated by measuring thyroid-stimulating hormone (TSH), free triiodothyronine (FT3), and free thyroxine (FT4). Bone turnover markers, including serum cross-linked C-telopeptide of type I collagen (CTX), parathyroid hormone (PTH), osteocalcin, vitamin D, and calcitonin, were measured using electrochemiluminescence immunoassay (ECLIA) methods on a Cobas e601 analyzer (Roche Diagnostics, Burgess Hill, UK), using commercial assay kits (CTX: cat. no. 11972308122; vitamin D: cat. no. 07464215190; osteocalcin: cat. no. 12149133122; PTH: cat. no. 11972103122; calcitonin: cat. no. 06445853190) [41]. To assess electrolyte balance, plasma levels of calcium, phosphorus, magnesium, and potassium were determined using standard commercial assays on a Roche Cobas C501 Chemistry Analyzer (Roche Diagnostics, Mannheim, Germany). Calcium concentrations were measured using an ion-selective electrode (ISE) method. Phosphorus and magnesium were quantified using photometric assays, while potassium levels were assessed by measuring the electromotive force (EMF) generated across a selective membrane, according to the manufacturer’s instructions. Plasma levels of gastrointestinal hormones, including Peptide YY (PYY), gastric inhibitory polypeptide (GIP), glucagon-like peptide-1 (GLP-1), and glucagon-like peptide-2 (GLP-2), were measured using commercially available enzyme immunoassay kits from Millipore (Merck Millipore, Darmstadt, Germany), based on chemiluminescent detection. The specific kits used were: PYY (EZGRT-89K), GIP (EZHGIP-54K), GLP-1 (EZGLPHS-35K), and GLP-2 (EZGLP-237K). Hormone levels were measured in duplicate from plasma samples as previously described [18,38,42,43].

Finally, molybdenum and iodine concentrations were determined according to validated analytical methods previously described [39,44]. Baseline comparability between the Control and Biofortified groups was verified using individual-level T0 data. No significant differences were observed between groups at baseline. Therefore, aggregated T0 values presented in Table 2 reflect the combined baseline characteristics of the study population.

### 2.4. Statistical Analysis

Statistical analysis was performed using GraphPad Prism 10 software (GraphPad Software, Dotmatics, Boston, MA, USA). Group × time interaction analyses could not be performed because baseline (T0) summary data were available only in aggregated form for the full sample and were not stored separately for each intervention group. This precluded the reconstruction of group-specific baseline distributions required for repeated-measures interaction modeling. Normality was assessed using the Shapiro–Wilk test and visually inspected through Q–Q plots (see Supplementary Materials). Differences in baseline characteristics between the two groups were evaluated using an unpaired *t*-test. Comparisons between groups, and within groups across time points, were performed using one-way ANOVA followed by Tukey’s post hoc test. When assumptions of normality were violated, appropriate non-parametric tests were considered Data are expressed as mean ± standard deviation (mean ± SD). Statistical significance was considered for *p* values ≤ 0.05. Regarding the Physical activity (PA), the protocol ensured that each administration primarily assessed physical activity during distinct, sequential 7-day windows of the study. The resulting data (number of days) were treated as an ordinal variable. The primary outcome was the change in self-reported physical activity, which was analyzed by comparing the mean scores across the three points (Baseline, Mid-Intervention, and Post-Intervention) using a Repeated Measures ANOVA (or the non-parametric equivalent, Friedman’s test) to determine if the changes in active days over the intervention period were statistically significant. No changes were observed (see Supplementary Materials).

## 3. Results

No significant changes in BMI, body weight, muscular fat mass and visceral fat (Table 2) emerged within the groups T0 and T1.

Daily consumption in the Biofortified group shows a beneficial effect on lipid metabolism. High-density lipoprotein (HDL) cholesterol significantly increased in the Biofortified group (49.86 ± 9.67 to 62.06 ± 14.81 mg/dL, *p* = 0.0025), while triglyceride levels significantly decreased (88.11 ± 18.58 to 63.38 ± 16.69 mg/dL, *p* = 0.0010). Consequently, the Biofortified group had significantly higher HDL and lower triglycerides than the Control group at T1 (*p* = 0.0048 and *p* = 0.0029, respectively). No significant within-group or between-group differences were observed for thyroid function (TSH), bone turnover markers (CTX, Osteocalcin, Vitamin D, PTH), or electrolyte balance (Calcium, Phosphorus, Magnesium, Potassium) following the intervention (Table 3).

The most pronounced effect was observed on the satiety hormone Peptide YY (PYY). Plasma PYY concentration nearly doubled in the Biofortified group, showing a significant increase from T0 to T1 (170.57 ± 55.30 to 266.48 ± 39.23 pg/mL, *p* = 0.0007). In contrast, levels in the Control group slightly decreased. At T1, PYY levels in the Biofortified group were significantly higher than in the Control group (*p* = 0.0001, Table 4). No significant changes were observed for other gastrointestinal hormones (GIP, GLP-1, GLP-2).

## 4. Discussion

The process of biofortification enhances the nutritional profile of crops, and this offers a promising strategy for addressing nutrient deficiencies across different population groups. By enriching commonly consumed vegetables with essential vitamins and minerals, biofortification not only provides a more nutrient-dense diet but also supports overall health and wellbeing [45,46,47]. In our study, foliar spray is the most effective fortification method since it ensures that the mineral is concentrated directly on the target plant. In contrast, fortifying the soil is unreliable, as we cannot control or accurately measure the amount the plant will absorb. This 12-day controlled nutritional intervention provides initial evidence that the daily consumption of 100 g of vegetables biofortified with iodine and 100 g biofortified with molybdenum are associated with acute, significant changes in specific metabolic biomarkers in a cohort of healthy adults aged 50–79 years, in late middle age and early older adulthood. The concentrations in the edible leaves as previously reported [37,38] of molybdenum were in control Lettuce (unfortified) 0.21 mg/100 g fresh weight while in Biofortified Lettuce (Mo-enriched) was 8 mg/100 g fresh weight while the concentrations of iodine in edible leaves were in control Lettuce (unfortified) 0.10 mg/100 g fresh weight while in Biofortified Lettuce (I-enriched) was 8.1 mg/100 g fresh weight. These values represent the true exposure levels and are substantially lower than the nominal amounts applied during cultivation. Based on the measured concentrations of Mo and I in the leaves, the estimated daily intake remained well below the established ULs for adults (1100 µg/day for iodine; 2 mg/day for molybdenum). Thus, the biofortified lettuce was safe for consumption within the duration of the trial.

The most relevant finding of this study is the significant improvement in key serum lipid and hepatic parameters following the consumption of biofortified lettuce. Specifically, the intervention was associated with a decrease in circulating triglycerides and liver enzymes (AST and ALT), together with an increase in HDL-cholesterol. All baseline values for these markers were within normal physiological ranges in a cohort of late middle aged and early older adulthood. The marked reduction in ALT and AST levels observed in the biofortified group within a relatively short timeframe should be interpreted with caution. However, several factors may have contributed to this finding: Iodine is essential for thyroid hormone production, which regulates metabolism and can influence liver function and Molybdenum acts as a cofactor for enzymes involved in detoxification, potentially improving liver health and function. Throughout the study, participants were instructed to maintain consistent dietary habits, and their intake was closely monitored. This control might have minimized confounding dietary factors that could have influenced liver enzyme levels. The consumption of biofortified foods may have provided a more nutrient-dense diet, which could have supported liver health and function. Therefore, the observed changes in ALT and AST levels may reflect acute physiological adaptations to dietary intervention in line with previous studies that have shown that dietary changes can lead to rapid alterations in metabolic biomarkers, particularly in response to dieting [48,49]. This suggests that the biofortified dietary intervention may exert a mild short-term modulatory effect even in the absence of pre-existing deficiency or dysfunction. Although both groups consumed the same amount and type of lettuce per day, only the biofortified group showed a marked increase in PYY. This observation must be interpreted with caution. At present, no established biological mechanism links iodine or molybdenum intake to acute stimulation of PYY secretion in humans, and the lettuce matrix itself was identical between groups.

While speculative indirect pathways, such as potential effects of iodine on gastrointestinal transit or possible microbiota-related influences of molybdenum, cannot be excluded, these mechanisms are unproven in humans and were not assessed in the present study. For these reasons, the PYY change is considered an exploratory finding that requires confirmation in larger, mechanistically designed trials. By demonstrating short-term variations in these biomarkers, this preliminary study provides a base for future extended research. The nutritional interventional daily consumption of 100 g of molybdenum-biofortified lettuce plus 100 g of iodine-biofortified lettuce—provides 8 mg of sodium molybdate and 1.21 mg of potassium iodate [41,44]. Safety considerations were evaluated with reference to the established Tolerable Upper Intake Levels (ULs) for adults (1100 µg/day for iodine; 2 mg/day for molybdenum). Based on the measured concentrations of these elements in the lettuce leaves, the estimated daily intakes during the intervention remained below these ULs [21]. Moisture content of the lettuce batches was also not analyzed, which may influence portion weight and satiety but cannot be quantified. Additionally, the short duration of the intervention makes body-weight changes unlikely and weight loss was not a study endpoint. A major limitation is the absence of group-specific T0 datasets, which prevented the use of repeated-measures ANOVA with a group × time interaction. As a result, temporal differences cannot be interpreted as intervention effects. To minimize bias the study (1) did include a control group receiving non-biofortified lettuce with identical handling, cultivation conditions, and post-harvest treatment. The only difference between the two interventions was the biofortification process itself. This design allowed us to directly attribute observed changes to the biofortified product. (2) The 12-day intervention and sample size of 36 participants were based on previous controlled-feeding trials investigating acute or short-term metabolic responses to functional foods. While not intended to assess long-term clinical outcomes, this design was appropriate for evaluating the biochemical and physiological responses associated with micronutrient-enriched foods under tightly controlled conditions. Power calculations confirmed sufficient sensitivity for detecting the targeted short-term metabolic changes. (3) Participants’ total dietary intake was carefully monitored throughout the study. Daily food records and dietary recalls were collected and analyzed to ensure that no significant differences existed between groups in energy or micronutrient intake apart from the experimental intervention. We implemented rigorous monitoring of participants’ dietary intake and lifestyle factors throughout the study to control potential confounding variables. This included maintaining consistent dietary habits and physical activity levels, as well as systematic tracking of food diaries and dietary recalls. (4) The biochemical parameters (lipid profile, liver enzymes, PYY, etc.) were selected as indicators of metabolic regulation and nutrient utilization. These markers provide insight into the early metabolic effects of biofortified foods and help characterize short-term physiological responses to micronutrient-enriched diets. (5) Our conclusions have been formulated cautiously to emphasize that the observed metabolic changes reflect short-term adaptations, rather than evidence of long-term disease prevention. We acknowledge that longer-term studies will be needed to confirm these effects. The strength of this study is its rigorous design: a randomized, single-blind, controlled trial conducted under tightly regulated laboratory conditions. The use of a whole food (lettuce) as the delivery matrix for the micronutrients enhances the translational relevance of the findings, as it reflects a realistic dietary intervention. To the best of our knowledge, no studies have investigated whether two or more molecules contained in a vegetable matrix biofortification can act synergistically and could achieve similar outcomes to those observed with individual biofortification after 12 days of daily ingestion in human population. Moreover, there are no studies that have investigated the effect of double-biofortification in healthy population. Previous research has also highlighted the relevance of maintaining adequate molybdenum and iodine status [33,34].

## 5. Conclusions

This study evaluated the short-term effects of daily consumption of iodine and molybdenum biofortified lettuce on metabolic biomarkers in a sample of healthy participants aged between 50 and 79 years. The findings indicate that 12-day consumption of 100 g of molybdenum-biofortified lettuce and 100 g of iodine-biofortified lettuce was associated with significant short-term changes in blood biomarkers of healthy participants aged between 50 and 79 years. The age range 50–79 was chosen because it presents relatively stable metabolic profiles, a higher prevalence of mild metabolic alterations relevant to the study endpoints, and lower day-to-day variability in biochemical markers compared with younger adults. This range also reflected the population’s most willing and able to adhere to a controlled 12-day dietary intervention. The intervention was associated with decreased plasma levels of triglycerides, AST, and ALT, and increased plasma levels of HDL-cholesterol and the satiety hormone PYY.

Participants’ feedback showed that the lettuce taste was well tolerated, with no negative comments reported. The lettuce was provided at no cost to participants as part of a collaborative project with the Department of Agricultural, Food and Forest Sciences at the University of Palermo. The fortification procedure and protocol were developed by this department in close collaboration with the Sicilian Department of Agriculture to advance new agricultural interventions. These results were achieved using physiological amounts of micronutrients delivered through a vegetable matrix, in accordance with the findings of previous studies in a healthy, young population. These aspects also align with broader sustainability principles, offering a complementary approach to conventional supplementation or food fortification. This study may serve as a base for future research to validate these findings over a longer period in a larger cohort. The findings support biofortification as a promising approach that warrants further investigation in longer-term studies.

## Figures and Tables

**Table 1:** Summary of the inclusion and exclusion criteria.

Inclusion Criteria	Exclusion Criteria
Age between 50 and 79 years	Presence of inflammatory chronic disease
Italian nationality	Use of medication, dietary supplements, and topical products
BMI between 18.5 and 28 kg/m^2^	Presence of viral infection
Absence of chronic age-related disease	Presence of blood-related dysfunction

BMI = body mass index.

**Table 2:** Participant anthropometric measurement before (T0) and after (T1) the 12-day intervention in the Control and Biofortification groups. T0 values reflect aggregated baseline data for the entire sample. Group-specific baseline summaries were not preserved in tabular form, but baseline comparability between groups was verified through statistical testing on the individual-level dataset before aggregation. Results are expressed as mean ± standard deviation (SD) and the (n) number of replicates for each experimental parameter determined.

Parameter	T0 All n = 36	T1 Control Group n = 18	T1 Biofortification Group n = 18
Weight (kg)	71.65 ± 13.55	70.24 ± 8.01	73.12 ± 21.92
Height (cm)	167.96 ± 5.03	169.29 ± 9.59	164.75 ± 12.90
Body Mass Index (BMI) (kg/m^2^)	25.15 ± 2.36	24.74 ± 1.87	25.66 ± 4.88
Fat mass (%)	27.68 ± 7.35	27.84 ± 7.73	27.55 ± 9.72
Muscular mass (%)	32.50 ± 5.50	33.21 ± 5.56	32.88 ± 7.02
Visceral fat (%)	7.83 ± 3.47	7.00 ± 3.00	7.91 ± 4.76
Basal metabolic rate (kcal)	1519 ± 242	1543 ± 177	1595± 359
Chest circumference (cm)	92.14 ± 9.00	91.75 ± 6.79	93.71 ± 13.21
Waist circumference (cm)	82.40 ± 11.21	79.69 ± 8.91	81.08 ± 12.37
Abdomen circumference (cm)	86.47 ± 9.05	87.07 ± 8.11	86.91 ± 10.71
Hip circumference (cm)	99.09 ± 6.96	99.88 ± 7.40	97.91 ± 7.06

**Table 3:** Hematological, liver and lipid profiles before (T0) and after (T1) the 12-day intervention in the Control and Biofortification groups. * denotes significant differences among groups (*p* < 0.05). Results are expressed as mean ± standard deviation (SD) and the (n) number of replicates for each experimental parameter determined.

Parameter	T0 All	T1 Control	T1 Biofortification	*p* Value #	*p* Value +	*p* Value §	Physiological Range
LDL (mg/dL)	100.31 ± 19.05	98.99 ± 25.10	96.15 ± 23.78	0.5104	0.8161	0.9088	100–129
HDL (mg/dL)	49.86 ± 9.67	49.89 ± 6.39	62.06 ± 14.81 *	0.9999	0.0025	0.0048	40–65
Total Chol (mg/dL)	182.46 ± 38.88	178.33 ± 26.01	170.50 ± 22.91	0.0653	0.9154	0.5580	120–200
Triglycerides (mg/dL)	88.11 ± 18.58	88.88 ± 21.10	63.38 ± 16.69 *	0.9921	0.0010	0.0029	50–150
Vitamin D (µg/L)	27.17 ± 10.41	28.69 ± 7.76	32.13 ± 9.17	0.9914	0.6232	0.9150	30–100
CTX (µg/L)	0.42 ± 0.15	0.42 ± 0.14	0.51 ± 0.17	0.9999	0.4400	0.5170	M 0.2–0.7 F 0.14–0.58
PTH (ng/L)	35.15 ± 11.44	32.60 ± 13.86	31.14 ± 7.28	0.9551	0.8241	0.9971	15–65
Osteocalcin (µg/L)	21.52 ± 7.78	20.29 ± 6.06	21.91 ± 5.47	0.9649	0.9996	0.9503	M 24–70 F 14–42
TSH (mlU/L)	2.07 ± 0.94	2.18 ± 0.71	2.44 ± 0.90	0.9946	0.6865	0.9290	0.27–4.2
AST (U/L)	21.53 ± 9.07	19.78 ± 8.06	13.40 ± 3.84 *	0.7135	0.0009	0.044	0–50
ALT (U/L)	23.50 ± 11.74	24.78 ± 12.48	13.06 ± 4.48 *	0.9931	0.0024	0.0041	0–50
Calcium (mg/dL)	9.40 ± 0.32	9.33 ± 0.25	9.57 ± 0.21	0.9450	0.3300	0.1739	8.6–10.2
Calcitonin (ng/L)	3.49 ± 3.17	4.07 ± 8.67	2.33 ± 1.53	0.9217	0.9426	0.6483	0–9.82
P (mg/dL)	3.44 ± 0.48	3.30 ± 0.38	3.26 ± 0.36	0.8052	0.7442	0.9994	2.5–4.5
Mg (mg/dL)	2.12 ± 0.14	2.09 ± 0.10	2.08 ± 0.11	0.8769	0.7884	0.7884	1.6–2.5
K (mmol/L)	4.31 ± 0.38	4.05 ± 0.33	4.32 ± 0.54	0.1316	>0.9999	0.2500	3.3–5.1

LDL = low-density lipoprotein; HDL = high-density lipoprotein; Chol = Cholesterol; CTX = beta-C-terminal telopeptide; TSH = thyroid stimulating hormone; AST = Aspartate transferase; ALT = Alanine transaminase; P = phosphorus level; Mg = Magnesium level; K = Potassium level; M = male; F: female. * indicates significant differences; *p* Value # = *p* values of the comparisons between the values of the entire sample at T0 and the Control group at T1; *p* Value + = *p* values of the comparisons between the values of the entire sample at T0 and the Biofortification group at T1; *p* Value § = *p* values of the comparisons between the values of the Control group at T1 and the Biofortification group at T1. Results are expressed as mean ± standard deviation (SD) and the (n) number of replicates for each experimental parameter determined.

**Table 4:** Gastrointestinal hormones levels before (T0) and after (T1) the 12-day intervention in the Control and Biofortification groups. * denotes significant differences among groups (*p* < 0.05). Results are expressed as mean ± standard deviation (SD) and the (n) number of replicates for each experimental parameter determined.

Parameter	T0 All	T1 Control	T1 Biofortification	*p* Value T0 All vs. T1 Control	*p* Value T0 All vs. T1 Biofortification	*p* Value T1 Control vs. T1 Biofortification
PYY (pg/mL)	170.57 ± 55.30	147.07 ± 47.76 *	266.48 ± 39.23 *	0.6589	0.0007	0.0001
GIP (pmol/L)	79.34 ± 49.05	71.31 ± 37.25	78.88 ± 29.91	0.9457	0.9999	0.9932
GLP-1 (pg/L)	6.23 ± 1.16	6.25 ± 0.90	5.14 ± 0.93	0.9999	0.1481	0.1690
GLP-2 (ng/L)	2.59 ± 0.80	2.63 ± 0.60	2.86 ± 0.98	0.9996	0.9252	0.9652

PYY = total Peptide YY; GIP = total Gastric inhibitory polypeptide; GLP-1 = Glucagon-like peptide-1; GLP-2 = Glucagon-like peptide-2.

## Data Availability

Data available in a publicly accessible repository: The original data presented in the study are openly available at https://github.com/ccortis/ accessed on 5 September 2025.

## Supplementary Materials

The following supporting information can be downloaded at: https://www.mdpi.com/article/10.3390/nu18010002/s1.

## Abbreviations

The following abbreviations are used in this manuscript:

T3	Triiodothyronine
T4	Thyroxine
RDA	Recommended Dietary Allowance
BMR	Basal Metabolic Rate
mARC	Mitochondrial Amidoxime Reducing Component
BEs	Biomonitoring Equivalents
NAFLD	Non-Alcoholic Fatty Liver Disease
HCC	Hepatocellular Carcinoma
TNF-α	Tumor Necrosis Factor-alpha
IL-6	Interleukin-6
IL-1β	Interleukin-1 beta
CVD	Cardiovascular Disease
NABbio	Nutrition, Age, and Bone (laboratory)
STEBICEF	Department of Biological, Chemical and Pharmaceutical Sciences and Technologies
BMI	Body Mass Index
T0	Before the intervention
T1	After the intervention
K	Potassium
ALB	Albumin
ALP	Alkaline Phosphatase
ALT	Alanine Transaminase
Ca	Calcium
HDL	High-Density Lipoprotein
CHO	Cholesterol
MG	Magnesium
FT3	Free Triiodothyronine
FT4	Free Thyroxine
TSH	Thyroid-Stimulating Hormone
TRIGL	Triglycerides
CTX	C-telopeptide of type I collagen
PTH	Parathyroid Hormone
ECLIA	Electro-chemiluminescence Immunoassay
P	Phosphorus
SD	Standard Deviation
PYY	Peptide YY
GIP	Gastric Inhibitory Polypeptide
GLP-1	Glucagon-like Peptide-1
GLP-2	Glucagon-like Peptide-2
LDL	Low-Density Lipoprotein
