# Supplementary material for: Biofortification of Vegetables with Iodine and Molybdenum for Healthy Nutrition: A Controlled Trial
Source: Nutrients. 2025 Dec 19;18(1):2. doi: 10.3390/nu18010002 (PMC12787933; doi:10.3390/nu18010002)
Supplement: Supplementary file 1 [file nutrients-18-00002-s001.zip › questionario informativo Studio lattuga biofortificata 2023.pdf]

Richiesta partecipazione e questionario informativo Studio lattuga biofortificata 2023

36 risposte

Dati anagrafici

Nome

Copia

36 risposte

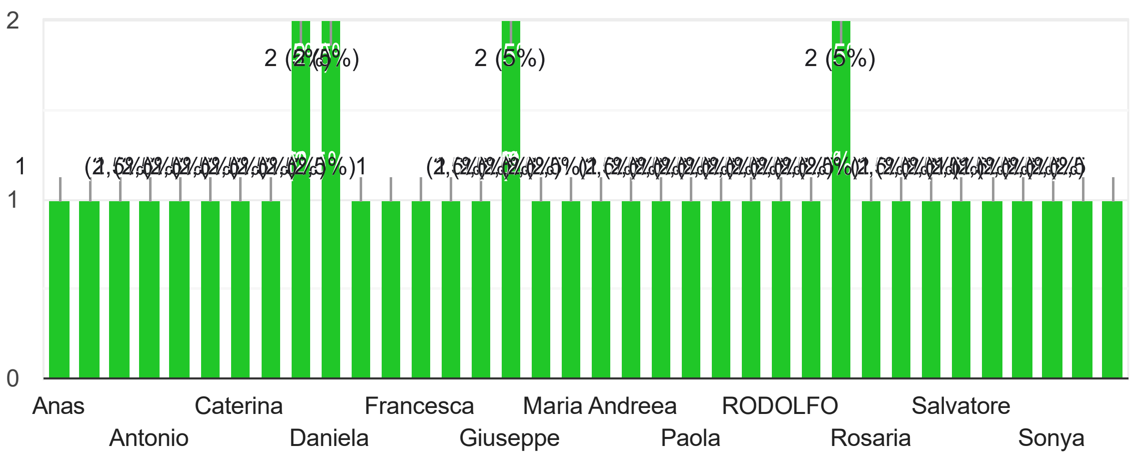

Cognome

Copia

36 risposte

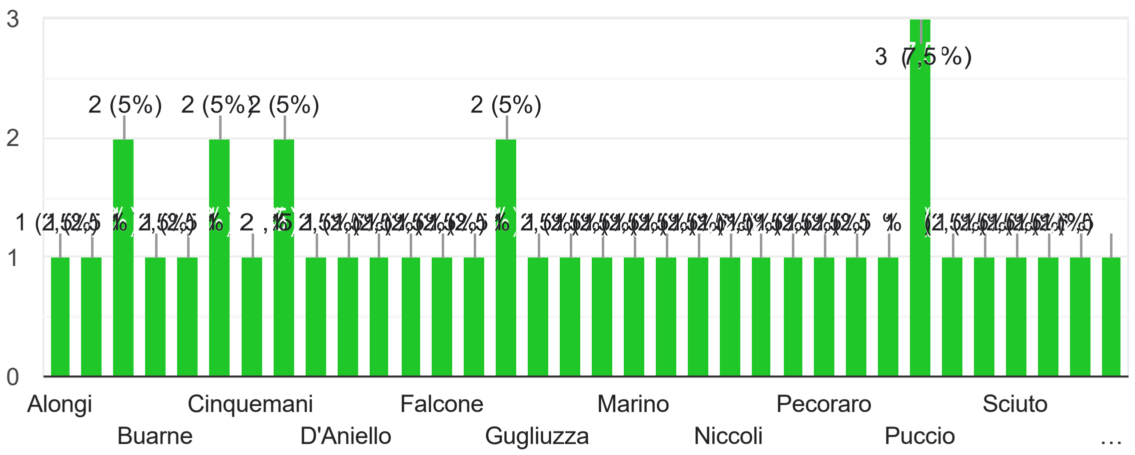

Sesso

Copia

36 risposte

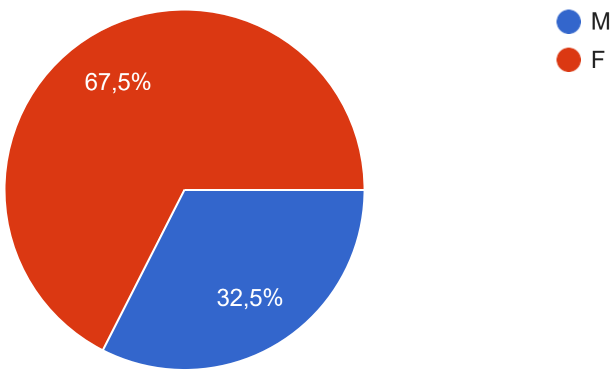

Età

Copia

36 risposte

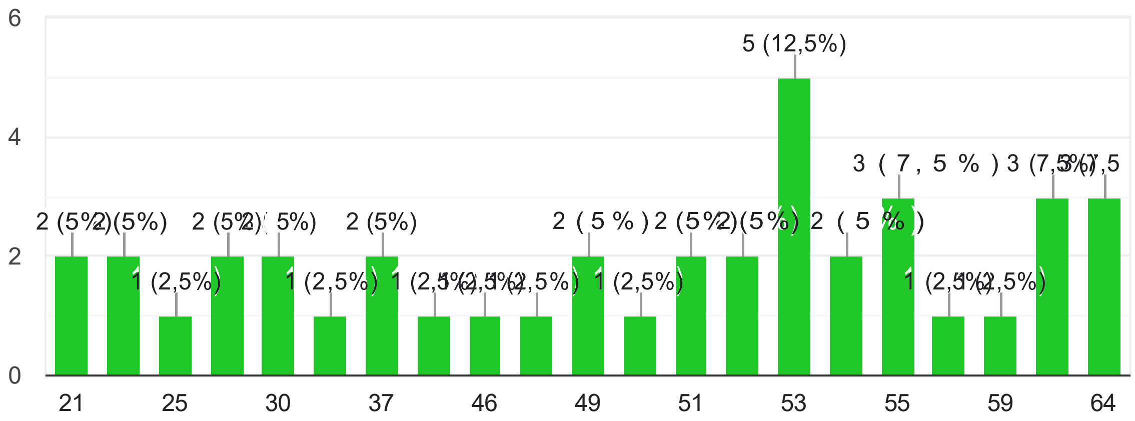

Data di nascita

36 risposte

nov 1958	7
dic 1958	16
gen 1959	3
ago 1952	25
nov 1962	23
dic 1952	22
ago 1943	29
mar 1966	8
giu 1947	14
ago 1947	29
mar 1958	5
set 1958	1
apr 1959	4
lug 1959	2 2
ott 1969	16
mar 1973	27
ago 1963	12
mag 1965	22
apr 1966	28
feb 1945	21
feb 1943	18
ago 1945	19
mar 1956	18
giu 1967	24
ago 1946	19
mar 1958	15

set 1954	9
apr 1951	3
lug 1959	8
ott 1969	11
mar 1973	25
ago 1963	7
mag 1955	28
apr 1946	28
feb 1945	1
feb 1942	3

Numero di cellulare

Copia

36 risposte

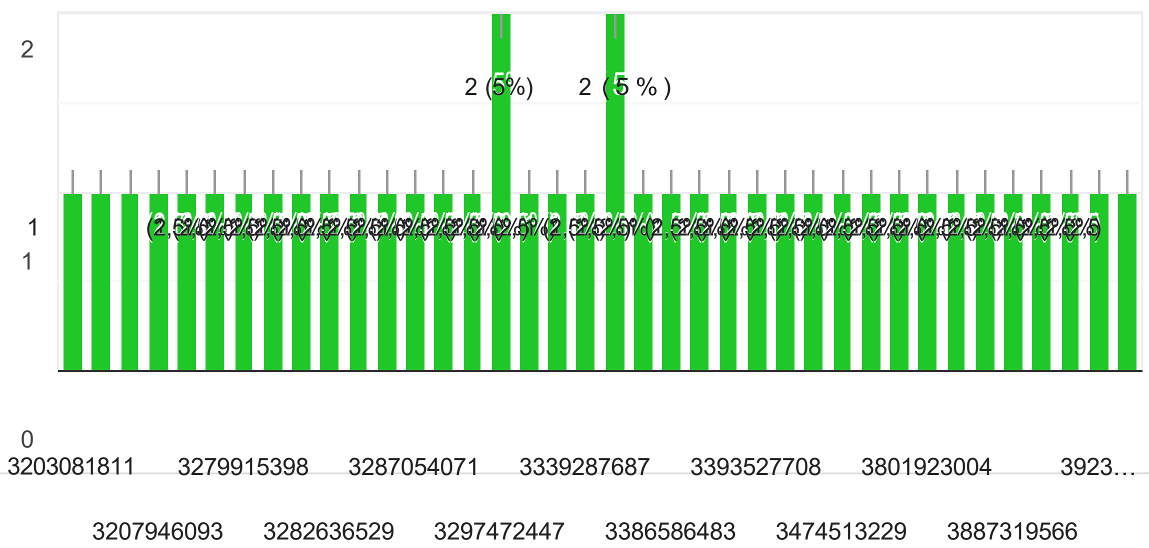

E-mail

Copia

36 risposte

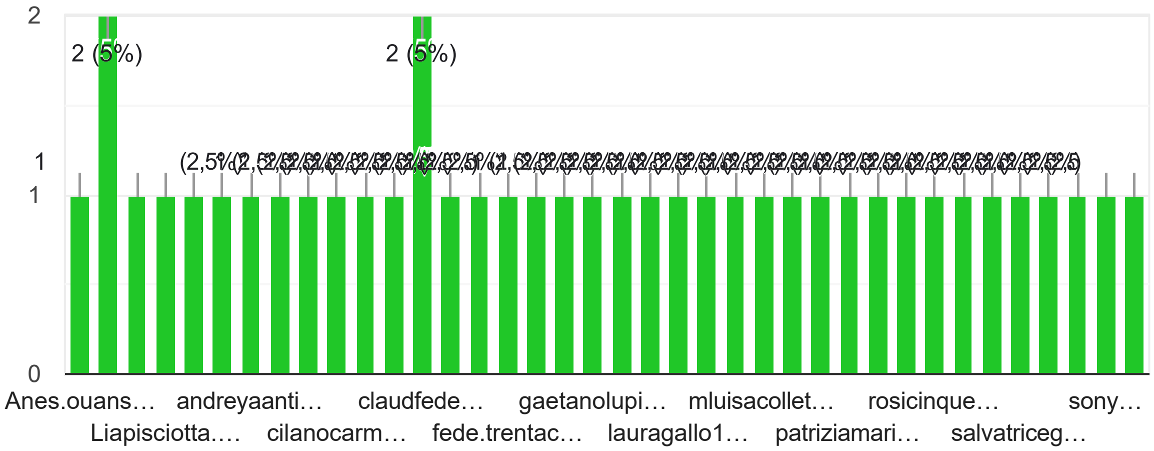

Anamnesi

Sta assumendo farmaci? Se sì, quali (es. Metformina, antistaminici)

36 risposte

No

no

No

NO

Nobisrar si

Terazosina, Triatec

nessuno

Metformina

Antiipertensivi, antistaminici, vit. D, cardioaspirina.

Omeprazolo

per ipertensione - enapren - moduretic - norvasc

Actonel , Dibase(vitamina D)

NO

BETABLOCCANTE LOBIDIUR 5MG/12,5MG PER REGOLARE BATTITO CARDIACO

Eutirox

Nebilox 5mg, Ipstyl 60mg (lanreotide) Di base 25.000

METHOTREXATE

Nebilox 5mg, Ipstyl 60mg(lanreotide)

Non assumo farmaci

Sta assumendo integratori alimentari e/o sportivi? Se sì, quali

36 risposte

No

no

NO

SOLO QUANDO FACCIO DOPPIO ALLENAMENTO OPPURE GARE AMINOACIDI

Creatina e aminoacidi

Colest tab plus

5grdi creatina ,8gr di aminoacidi

Si, Ellen

Un cucchiaino di magnesio supremo

Ansiodep

Creatina, multivitaminico, vitamina D, proteine in polvere

magnesio e potassio

Spirulina

Si, Flomel compresse

Ultramag (Magnesio) , Condrotin solfato sodico

Colecalciferolo DIBASE da 50.000 U.I./2,5 ml

Non assumo integratori

È andato incontro a grandi variazioni di peso negli ultimi 5 anni?

Copia

36 risposte

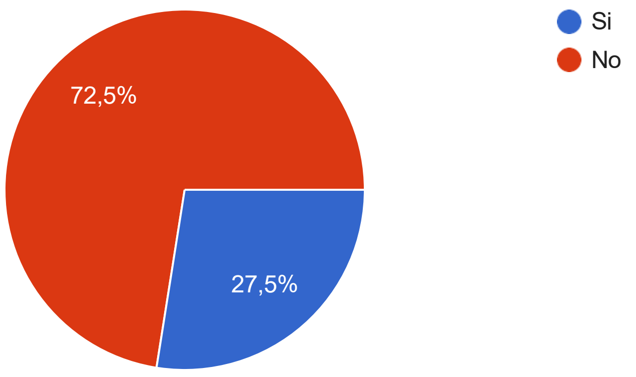

Ha subito interventi chirurgici negli ultimi 5 anni?

Copia

36 risposte

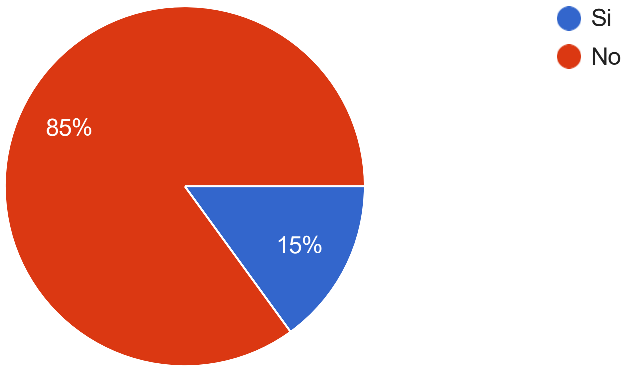

Soffre di patologie cardiovascolari? Se sì, quali (es. ipertensione, aritmia, ecc.)

36 risposte

No

no

NO

Quando sono un pò nervosa gni ranto aritmia...sporadicamente

Ipertensione e ipertrofia prostatica

nessuna

Vene varicose

Ipertensione

Insufficienza venosa

ipertensione

LEGGERA ARITMIA

Calcolosi renale

Ipertensione

Non stò soffrendo di nulla

Soffre di patologie all'apparato gastrointestinale? Se sì, quali (es. gastrite, reflusso gastrico, colite, morbo di Crohn o altre sindromi da cattivo assorbimento, ecc.)

36 risposte

No

no

NO

No

reflusso gastrico

ho avuto un caso di gastrite il mese scorso

nessuno

Gastrite e reflusso gastro esofageo

Gastrite

Reflusso gastrico e gastrite

Reflusso gastrico

UN POCO DI REFLUSSO GASTRICO

Un po' di gastrite

Ho la colite

Soffre di patologie alla tiroide? Se sì, quali (es. ipotiroidismo, ipertiroidismo, tiroidite autoimmune ecc.)

36 risposte

No

no

NO

No

nessuno

Ipotiroidismo

No soffro di tiroide

Soffre di patologie all'apparato renale? Se sì, quali (es. calcoli renali, insufficienza renale, nefropatia diabetica ecc.)

36 risposte

No

no

NO

nessuno

Calcoli renali

Non soffro di reni

Soffre di patologie autoimmuni? Se sì, quali? (es. celiachia, artrite reumatoide, morbo di Graves, vasculite, tiroiditi autoimmuni ecc.)

36 risposte

No

no

NO

nessuno

Psoriasi

Orticaria idiopatica

artrite reumatoide

Non soffro di niente

Soffre di anemia?

Copia

36 risposte

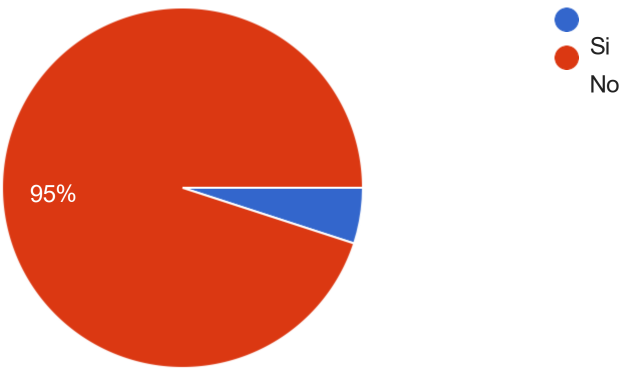

Soffre di qualche sindrome metabolica? Se sì, quale (es. diabete di tipo I/II, obesità, iperinsulinemia, ecc.)

36 risposte

No

no

NO

nessuna

odesità

SOVRAPPESO

Soffre di altri tipi di patologie non specificate in precedenza? Se sì, quali

36 risposte

No

no

NO

Acromegalia recidivata (adenoma ipofisario)

nessuno

Asma

È un soggetto fumatore?

Copia

36 risposte

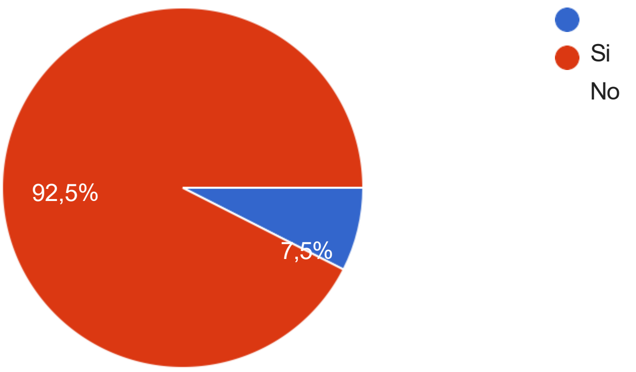

Pratica attività fisica? Se sì, quale (es. camminata, corsa, pesistica ecc.)

36 risposte

Camminata

no

camminata

Calisthenics

Total body

pilates e padel

Solo nel periodo estivo bicicletta

CORSA,BICI NUOTO ,TRAIL (GORSAV IN MONTAGNA) PESI

Allenamento corpo libero

Pesistica corsa

pesistica

Nuoto, ciclismo a livello amatoriale

Allenamento a corpo libero

Camminata, pilates

Pilates

Camminata

Sì, pesistica

allenamento funzionale

Palestra body pump/thai fit

Si.aerobica pesistica totalbody

Si, Total body calisthenics

Si, fitness

Allenamento funzionale

vela

Palestra corpo libero

CAMMINATA VELOCE, PILATES, BALLO CARAIBICO

Pesistica e corpo libero

Jump,allenamento a corpo libero e pesi

Pesistica ,allenamento a corpo libero e jump

Si palestra corpo libero

Attualmente faccio allenamento a colpo libero

si palestra corpo libero 3 volte a settimana

Quante volte a settimana fa sport?

Copia

36 risposte

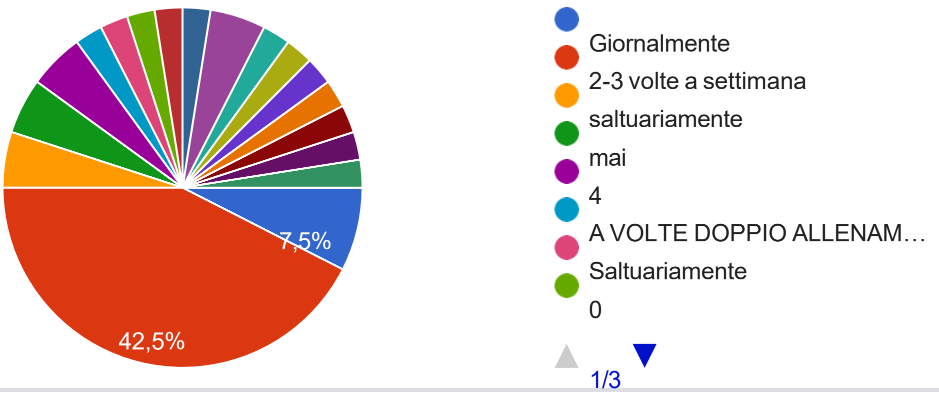

Quanto tempo al giorno dedica all'attività fisica?

Copia

36 risposte

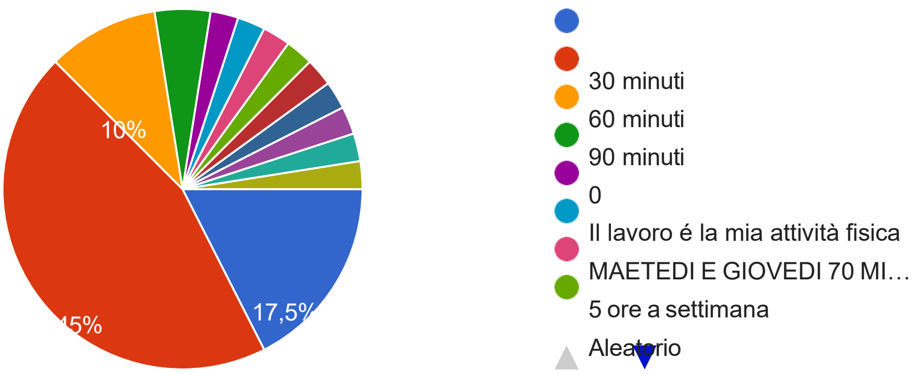

1/2

Da quanti anni pratica sport?

36 risposte

4 anni

0

8

1

20 anni

Fino a 18 anni tre ore e più al giorno

DA SEMPRE

Da piccolo

Quasi 30 anni

50

20

Da sempre

Da sempre

10

Mi sono fermata durante la pandemia ed ho ri da poco

4

saltuariamente

3 mesi

15 anni

16

22

25

da sempre

2

Non faccio sport

1 ANNO

10 anni

16 anni

mai praticato

Da 2

6 mesi

Non sono un tipo che faccio sport

12 mesi

Soffre di stipsi?

Copia

36 risposte

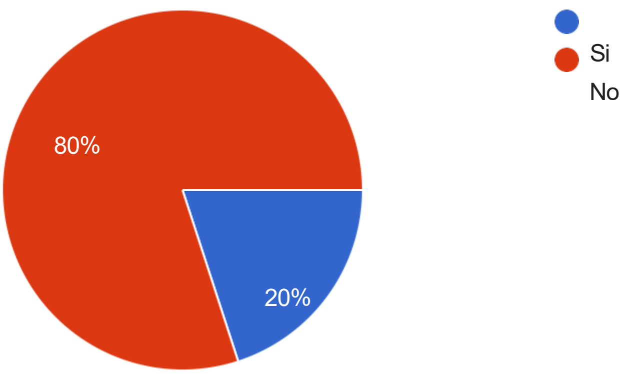

Soffre di allergie alimentari? Se sì, quali

36 risposte

No

no

NO

No

Nessuna

nessuna

Carciofi,fave

No

NON CHE IO SAPPIA

Soffre di intolleranze alimentari? Se sì, quali

36 risposte

No

no

Lattosio

Intollerante al lattosio

NO

No

nessuna

Alcuni Formaggi,pesce(orata,salmone)

Lievito di birra , latticini

NON SO

NON CHE IO SAPPIA

Albume ,latte di capre e pecora ,pisellini

uova e latticini

Non soffro di niente

Abitudini alimentari

Sta seguendo qualche tipo di regime alimentare particolare? Se sì indichi il tipo con eventuali specifiche se ritenute rilevanti (es. dieta iperproteica, ipocalorica, chetogenica, vegetariana, vegana ecc.).

36 risposte

No

no

Dieta ipocalorica

NO

nessuno

Digiuno intermittente

PIRIPICCHIO

No

Regolare alimentazione da dieta iperproteica

nessuna

Dieta iperproteica

Sì, dieta iperproteica e ipercalorica

digiuno intermittente (16 ore)

Regime equilibrato

Dieta mediterranea

Una dieta sana ed equilibrata

Dieta mediterranea

Non faccio dieta da tempo

Quanti pasti fa al giorno? (es. pranzo, spuntino, cena)

Copia

36 risposte

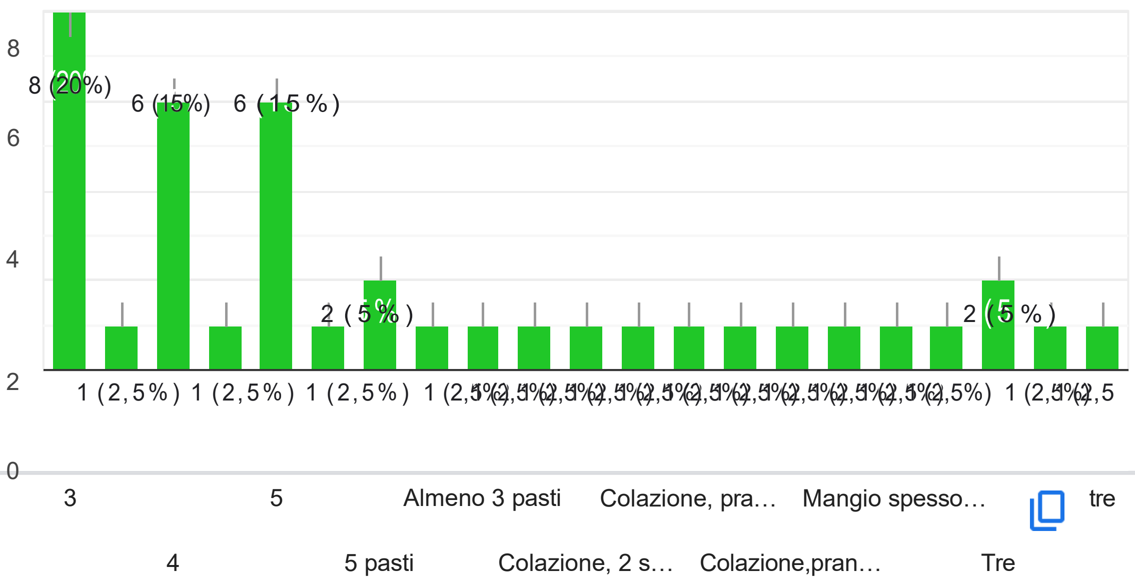

Salta la colazione?

Copia

36 risposte

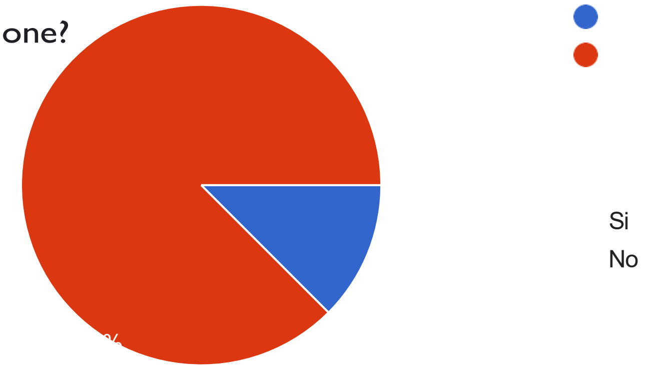

Quanti litri di acqua beve approssimativamente al giorno?

Copia

36 risposte

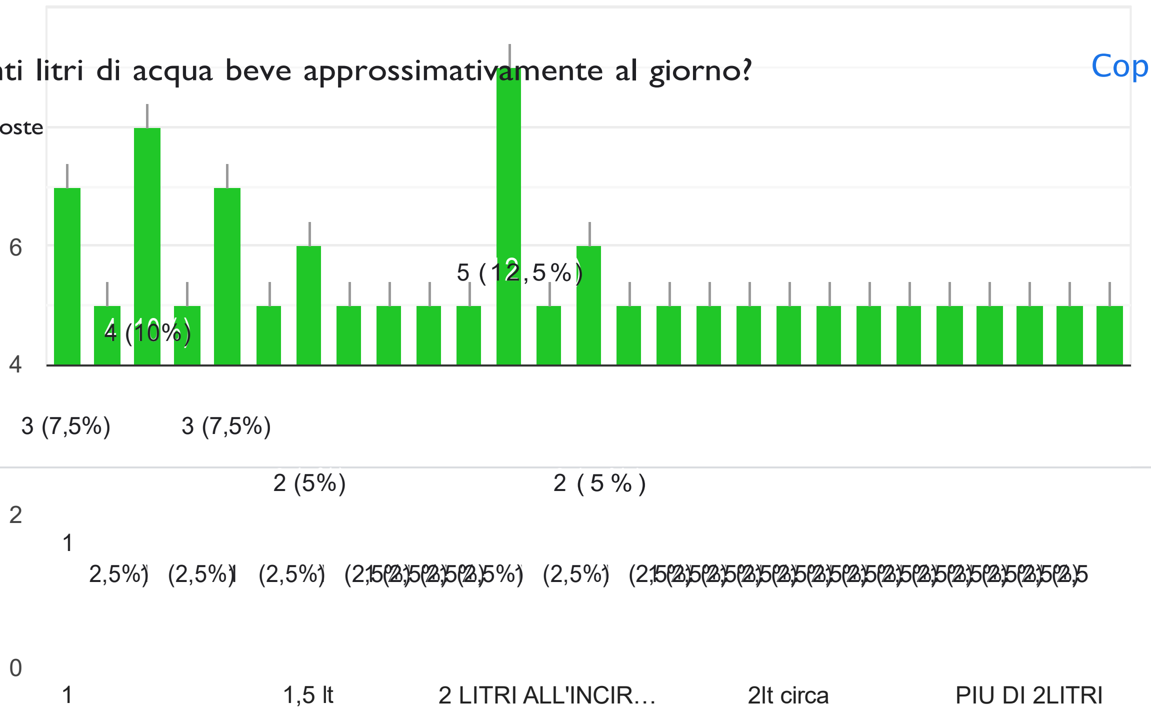

1 litro e mezzo	1/2	2,5	3/4 litri

Quante volte a settimana mangia pasta?

Copia

36 risposte

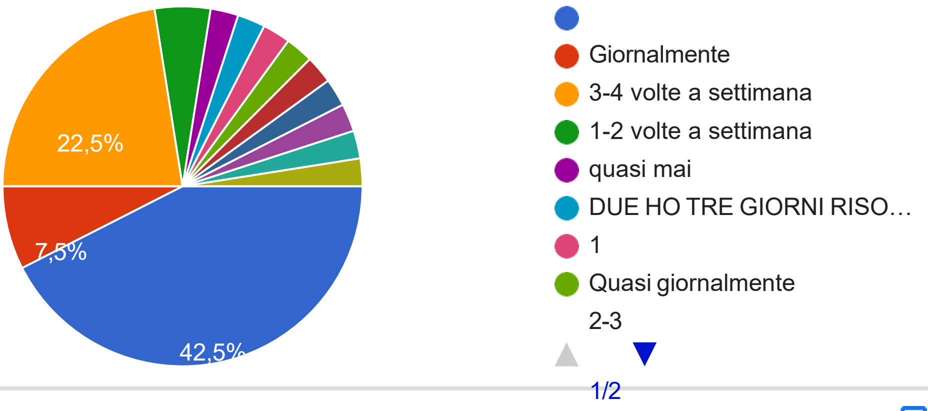

Quante volte a settimana mangia cereali? (es. orzo, farro, avena, quinoa, ecc.)

Copia

36 risposte

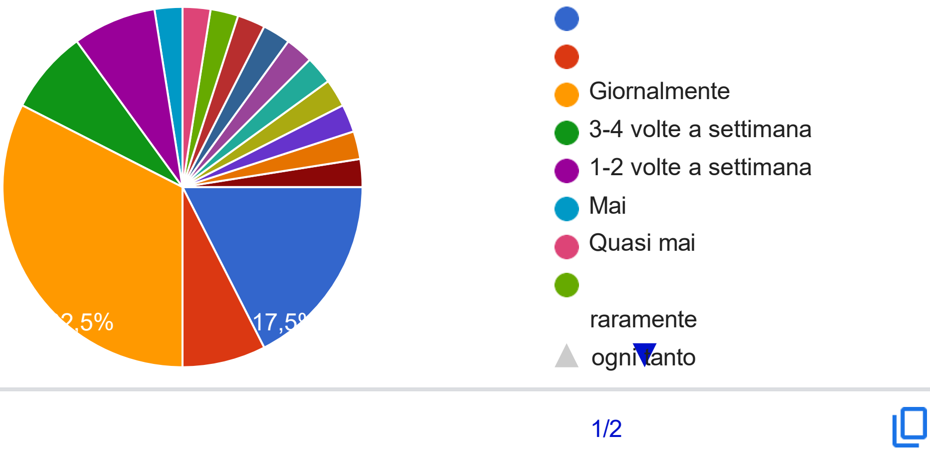

Quante volte a settimana mangia pane?

Copia

36 risposte

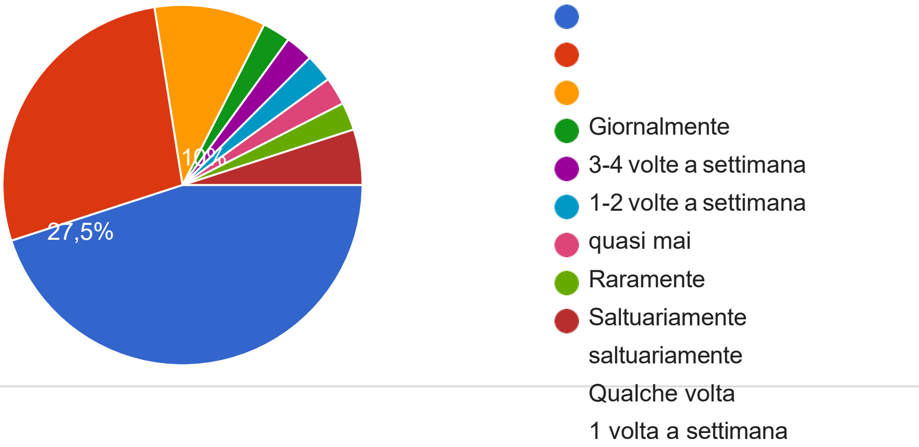

Quante volte a settimana mangia riso?

Copia

36 risposte

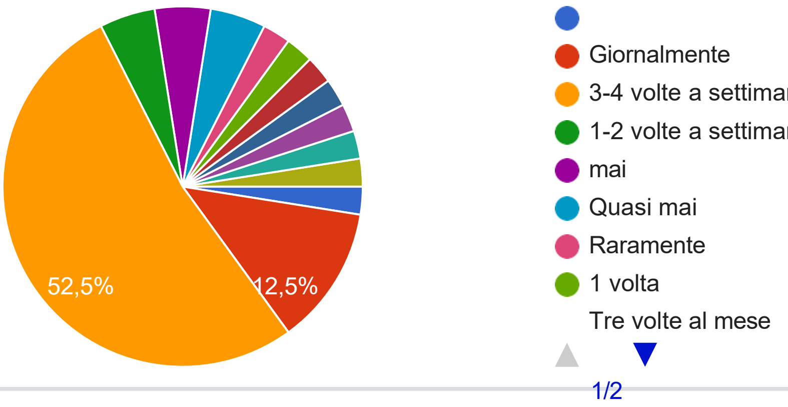

Quante volte a settimana mangia patate?

Copia

36 risposte

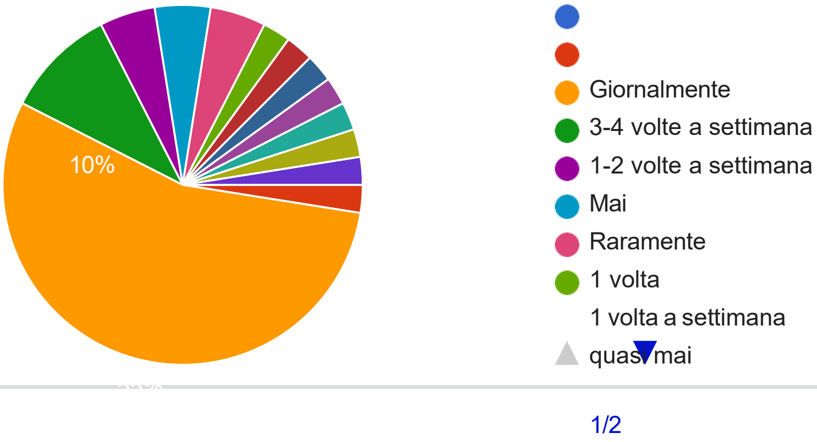

Quante volte a settimana mangia carne rossa?

Copia

36 risposte

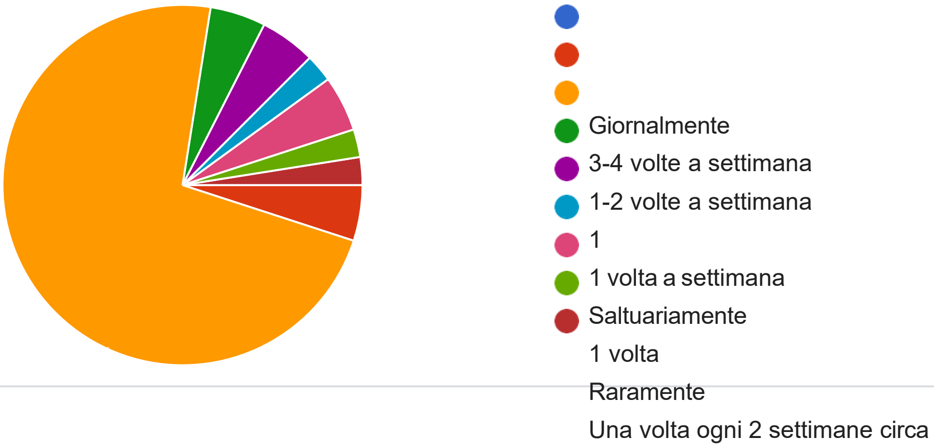

Quante volte a settimana mangia carne bianca?

Copia

36 risposte

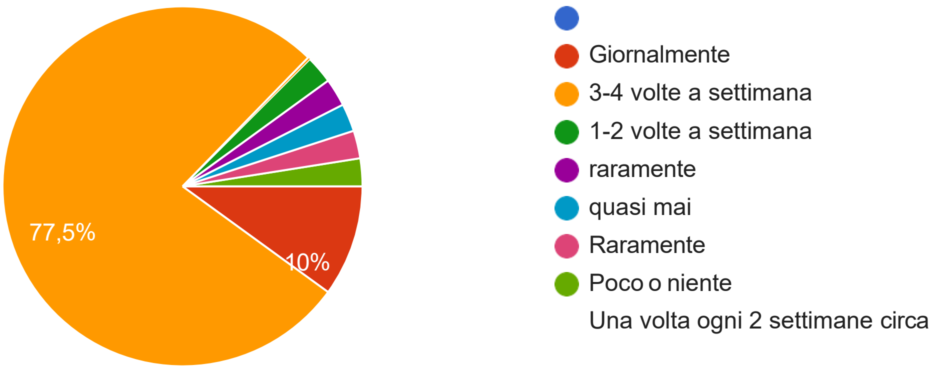

Quante volte a settimana mangia pesce?

Copia

36 risposte

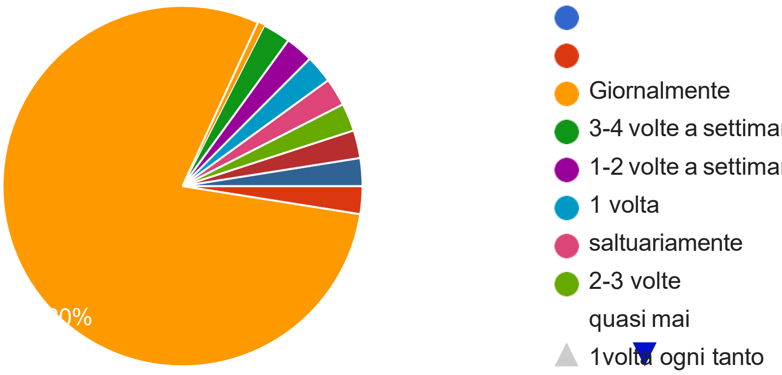

Quante volte a settimana mangia legumi?

Copia

36 risposte

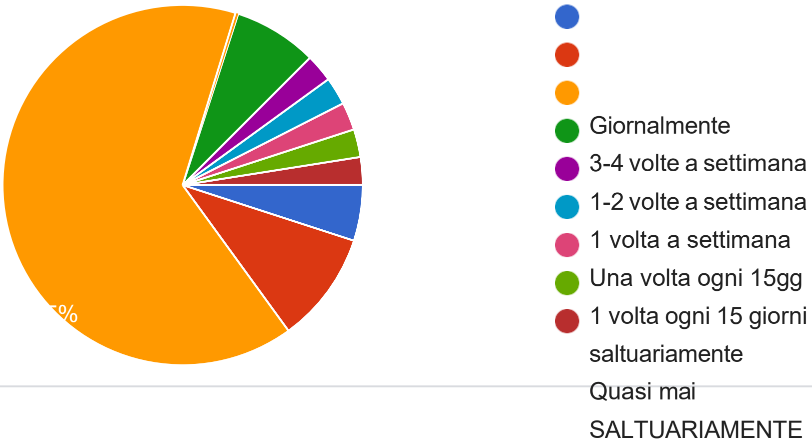

Quante volte a settimana mangia uova?

Copia

36 risposte

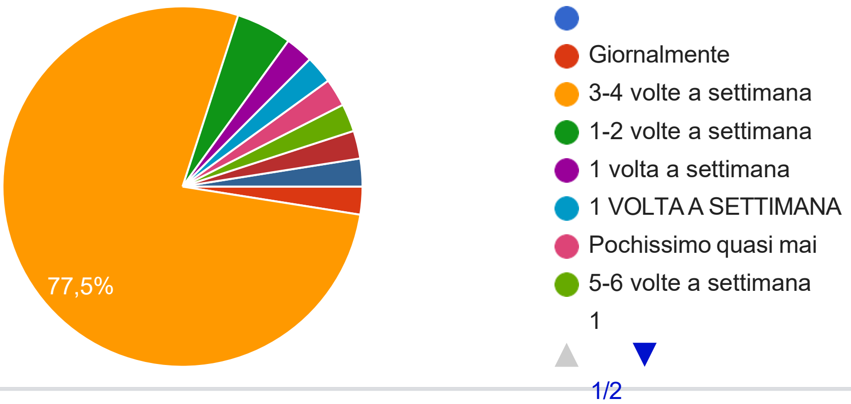

Quante volte a settimana mangia insaccati?

Copia

36 risposte

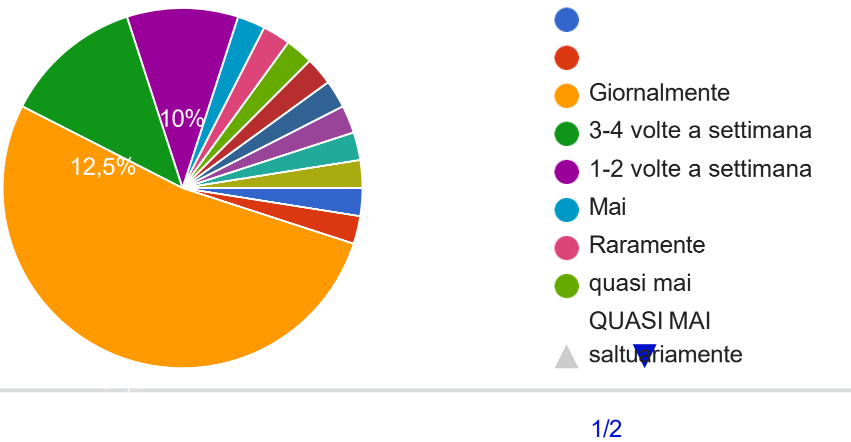

Quante volte a settimana mangia cibo in scatola?

Copia

36 risposte

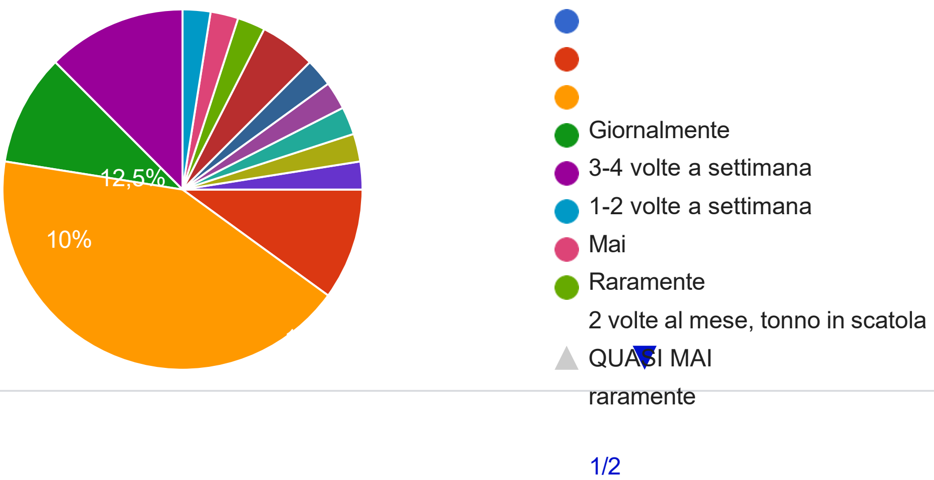

Quante volte a settimana mangia dolciumi (es.caramelle, cioccolatini, pasticcini ecc.)?

Copia

36 risposte

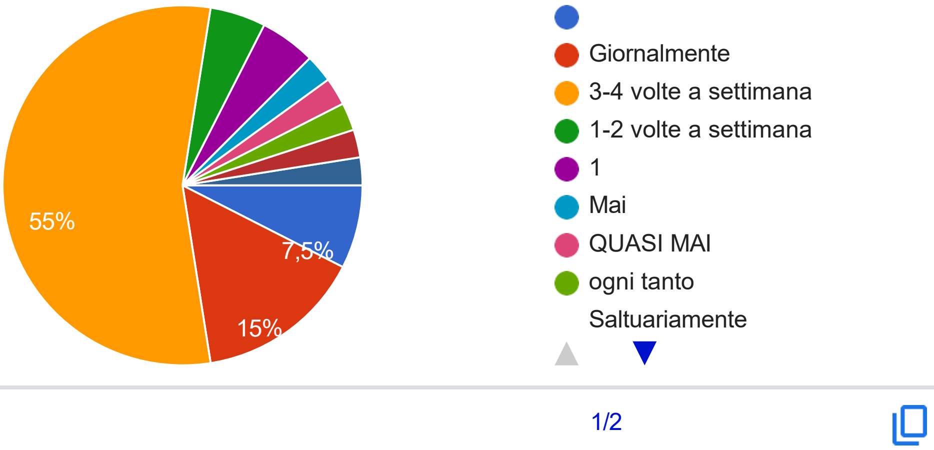

Quante volte a settimana mangia latte e/o latticini?

Copia

36 risposte

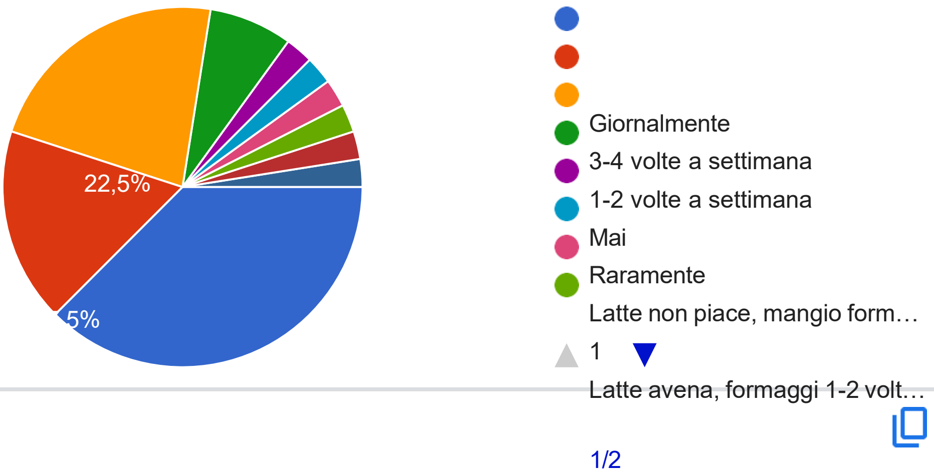

Quante volte a settimana mangia verdura?

Copia

36 risposte

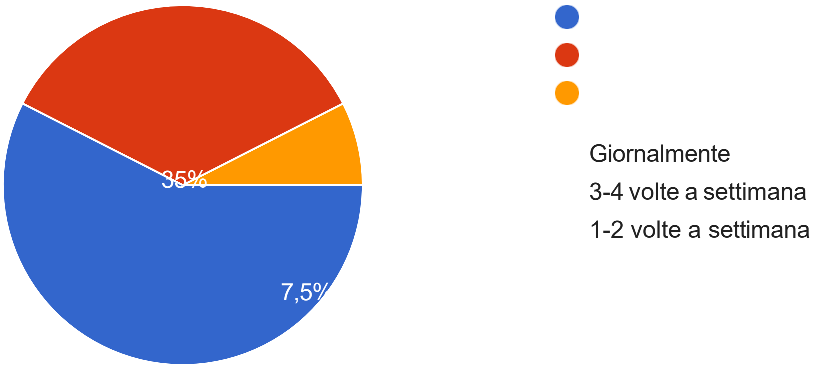

Quante volte a settimana mangia frutta?

Copia

36 risposte

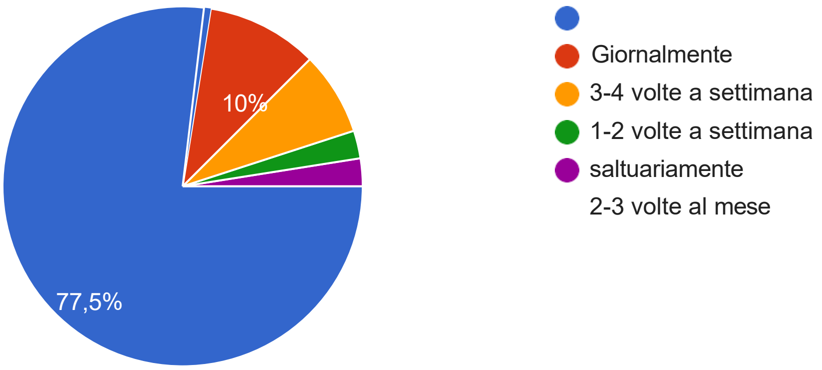

Quante volte a settimana assume bevande gassate e/o zuccherine?

Copia

36 risposte

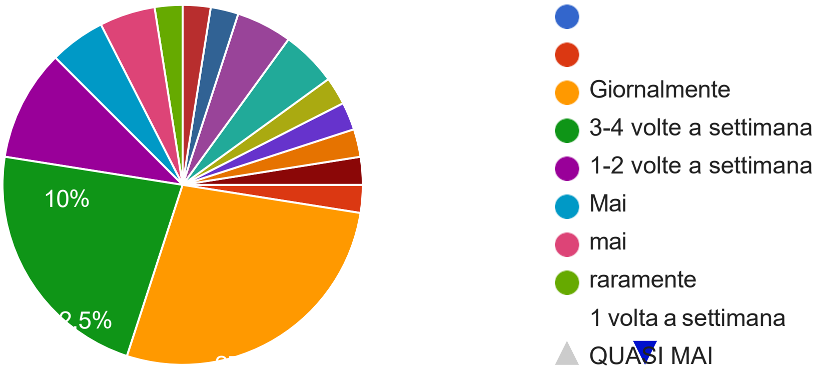

1/2

Quante volte a settimana assume alcolici?

Copia

36 risposte

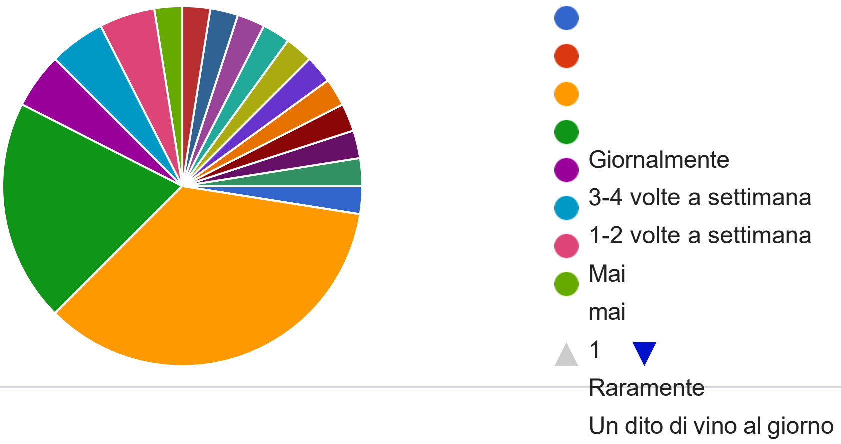

1/3

Quante volte a settimana mangia Junk-food? (cibo spazzatura: es. panini, pizza, patatine fritte ecc.)

Copia

36 risposte

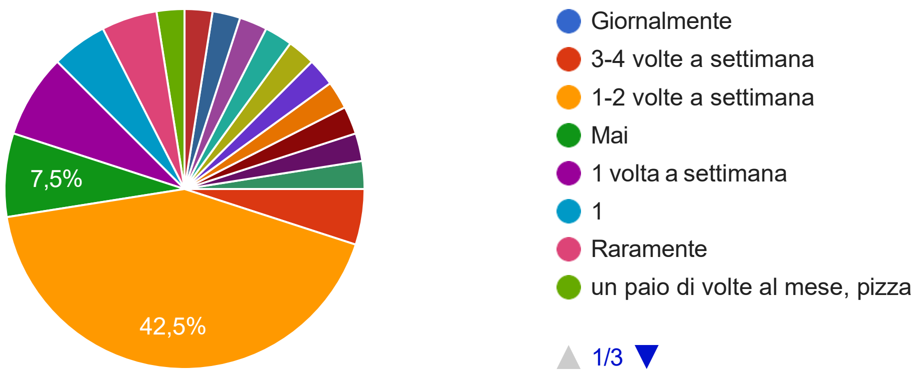

Quali dolcificanti utilizza? (zucchero raffinato bianco, di canna, dolcificanti chimici come saccarina o aspartame ecc.)

36 risposte

nessuno

Nessuno

zucchero di canna

Miele

Nulla

Mai

Zucchero raffinato bianco

Zucchero di canna

QUANDO VADO IN BICI ASSUMO SALI MINERALI CREDO CHE LI CI SIANO DEGLI ZUCCHERI

Poco a settima 1-2

Zucchero raffinato bianco

Zucchero di canna

Zucchero di canna e bianco

Aspartame

Zucchero di canna o miele

Nel caffè la mattina zucchero di canna

Zucchero bianco e di canna

Zucchero bianco

NESSUNO

NESSUNO, SOLO NEL CAFFE' SALTUARIAMENTE

Zucchero raffinato

mai

Zucchero

Quasi mai

Zucchero normale

zucchero bianco

Che tipo di sale utilizza?

Copia

36 risposte

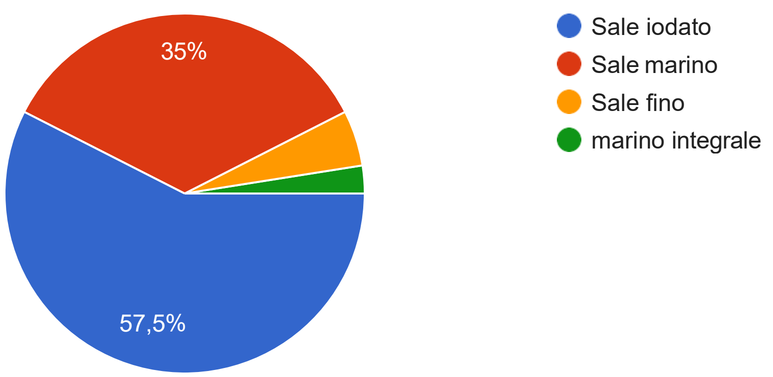

Grazie per la partecipazione

Questi contenuti non sono creati né avallati da Google. - Contatta il proprietario del modulo - Termini di servizio - Norme sulla privacy

Questo modulo sembra sospetto? Segnala

Google Moduli
