# Supplementary material for: Biofortification of Vegetables with Iodine and Molybdenum for Healthy Nutrition: A Controlled Trial
Source: Nutrients. 2025 Dec 19;18(1):2. doi: 10.3390/nu18010002 (PMC12787933; doi:10.3390/nu18010002)
Supplement: Supplementary file 1 [file nutrients-18-00002-s001.zip › Table Dietary items.pdf]

Table S1. Dietary assessment of subjects in the two groups (LC = lettuce control, LB = lettuce biofortified (100 g I-LB+100 g Mo-LB)) at baseline (T0) and following 12 day of lettuce administration (200 grams/day) (T1). All the values are indicated as means ± standard deviations (SD). Student t tests were used to compare two groups (LC = lettuce control, LB = lettuce biofortified (100 g I-LB+100 g Mo-LB) at baseline. When appropriate differences between and within the groups (T0 and T1) were compared by using one-way ANOVA followed by Tukey's posttest. A p-value higher than 0.05 means that the change is not statistically significant and reported as not statistically significant (n.s.).

**Dietary assessment**	**Control group T0** (n=36; 19 females; 17 males) mean ± S.D.	**Control group T1** (n=20; 8 females; 12 males) mean ± S.D.	**Biofortified lettuce group T1** (n=20; 9 females; 11 males) mean ± S.D.	**p-value**
**Energy intake (kcal/day)**	1894 ± 427	2001 ±357	1947 ± 387	n.s.
**Protein (g/day)**	67.5 ± 14	71 ± 22	70 ± 19	n.s.
**Carbohydrates (g/day)**	249 ± 52	263 ± 81	265 ± 41	n.s.
**Fats (g/day)**	71.5 ± 23.5	72 ± 31	68± 19	n.s.
**Cholesterol (mg/day)**	210± 104.5	207± 194	206± 34	n.s.
**Monounsaturated fatty acids (g/day)**	36.4 ± 11	31 ± 14	33 ± 34	n.s.
**Fibers (g/day)**	23 ± 3	17 ± 10	22 ± 3	n.s.
**Water (liter)**	1.7 ± 0.2	1.6 ± 0.4	1.7 ± 0.1	n.s.

Table S2. Self-Reported Physical Activity (PA) at Baseline and Post-Intervention. PA was measured using a Single-Item Measure (SIM) asking for the number of days (≥30 minutes of activity raising breathing rate) in the past week. Baseline (T0) data is presented for the entire sample before randomization. Post-Intervention (T1) data is presented by randomized group (Control vs. Biofortification).

Time Point	Group	N	Mean Days (0-7)	SD
**Baseline (T0)**	All Participants	36	2.0	1.0
**Post-Intervention (T1)**	Control Group	18	1.9	1.1
**Post-Intervention (T1)**	Biofortification Group	18	2.0	0.9
